# Geographical Divergence and Environmental Drivers of the Symbiotic Bacterial Community Structure in a *Koelreuteria*‐Feeding Aphid Species Complex

**DOI:** 10.1002/ece3.73580

**Published:** 2026-04-23

**Authors:** Xin Peng, Qian Liu, Qiang Li, Xiaolei Huang

**Affiliations:** ^1^ State Key Laboratory of Agricultural and Forestry Biosecurity, College of Plant Protection Fujian Agriculture and Forestry University Fuzhou China; ^2^ Fujian Provincial Key Laboratory of Insect Ecology Fujian Agriculture and Forestry University Fuzhou China

**Keywords:** 16S rRNA gene, environmental drivers, *Periphyllus koelreuteriae*, species complex, symbiotic bacteria

## Abstract

Symbiotic bacteria play a crucial role in the life history of insects. Aphids and their diverse symbiotic bacteria serve as an excellent model for studying the bacterial‐insect symbiotic relationship. Our recent study revealed that the aphid *Periphyllus koelreuteriae*, an important ornamental pest specifically feeding on *Koelreuteria* plants and widely distributed in the temperate and subtropical regions of China, is actually a species complex that includes three species (*P. koelreuteriae*, *P. blackmani*, and *P. guangxuei*). To characterize the composition and abundance of the symbiotic bacterial communities within this species complex, we employed Illumina NovaSeq high‐throughput sequencing to assess symbiotic bacterial diversity and further investigated the associations between symbiont community profiles and aphid species, geographic populations, and host plants. The results show that two dominant symbiotic bacteria were detected, namely *Buchnera* and *Serratia*. The mean relative abundance of *Buchnera* exhibited the trend: *P. guangxuei* (88.41%) < *P. blackmani* (95.36%) < *P. koelreuteriae* (98.51%), which are distributed in subtropical highland, subtropical humid, and temperate regions, respectively, whereas *Serratia* showed the opposite pattern. Redundancy analysis (RDA) revealed that latitude (LAT) and the minimum temperature of the coldest month (BIO6) are critical environmental factors affecting the composition of symbiotic bacteria in the *P. koelreuteriae* species complex. The relative abundance of *Buchnera* significantly decreased with decreasing latitude and increasing minimum temperature of the coldest month, whereas the relative abundance of *Serratia* exhibited the opposite. These results indicate that the composition and abundance of symbiotic bacteria in this species complex are influenced by both aphid species and geographic‐climatic conditions, with latitude (LAT) and the minimum temperature of the coldest month (BIO6) identified as key environmental factors shaping the community structure. This study elucidates the distribution patterns of symbiotic bacteria across closely allied aphid species and along environmental gradients, providing a theoretical foundation for understanding the ecological adaptation mechanisms of this aphid species complex and laying a scientific basis for developing targeted integrated management strategies in the future.

## Graphical abstract

This study investigates the symbiotic bacteria in the *Periphyllus koelreuteriae* aphid species complex, revealing an inverse relationship between the dominant symbionts *Buchnera* and *Serratia*. The relative abundance of these symbionts is strongly influenced by latitude and minimum winter temperature, suggesting that heat stress drives their differentiation. These findings provide key insights into aphid‐symbiont coevolution and its role in host adaptation.

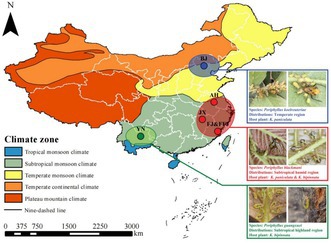

## 1 Introduction

*Periphyllus koelreuteriae* (Hemiptera: Aphididae: Chaitophorinae) is an important ornamental pest that specifically feeds on golden raintrees, the *Koelreuteria* plants (Takahashi 1919). Unlike most Chaitophorinae species confined to temperate regions, *P. koelreuteriae* is widely distributed in the temperate and subtropical regions of East Asia. The broad‐scale spatial distribution has facilitated the genetic differentiation of *P. koelreuteriae* in China. Li et al. (2021) conducted phylogenetic and haplotype network analysis using several genes, revealing that the Chinese populations of *P. koelreuteriae* have differentiated into three distinct genetic lineages. These lineages correspond to temperate, subtropical humid, and subtropical highland regions, respectively. The temperate lineage exclusively feeds on *K. paniculata* , the subtropical highland lineage exclusively feeds on *K. bipinnata* , while the subtropical humid lineage is capable of feeding on both *K. paniculata* and *K. bipinnata* simultaneously. Subsequently, Li et al. (2022) confirmed two cryptic species in China, *Periphyllus blackmani* and *P. guangxuei*, based on integrated morphological and genetic evidence. Therefore, *P. koelreuteriae* actually comprises a species complex consisting of three distinct species.

Symbiotic bacteria play a crucial role in the life history of aphids. Aphids and their diverse symbiotic bacteria serve as an excellent model for studying bacteria–insect symbiosis relationship (Liu et al. 2023, 2021). The survival and reproduction of aphids are inseparable from their obligate endosymbiont *Buchnera*, with which they share a strict obligate intracellular endosymbiotic relationship. In this mutualistic relationship, aphids provide *Buchnera* with a protected intracellular environment and essential nutrients, while *Buchnera* synthesize essential amino acids for aphid hosts that cannot be produced by the aphids themselves and are scarce in plant sap (Baumann 2005; Douglas 1998). *Buchnera* is ubiquitously present in almost all aphid species. It colonizes specialized organs within the aphid body known as bacteriocytes and is strictly vertically transmitted to offspring (Buchner 1965; Moran et al. 1993). Moreover, the phylogeny of *Buchnera* exhibits a high degree of congruence with that of its aphid hosts, reflecting a long‐term coevolutionary history between them (Clark et al. 2000; Moran et al. 1999). Furthermore, aphids often carry one or more species of facultative symbiotic bacteria. These facultative symbionts are typically located in secondary bacteriocytes, sheath cells, or hemolymph, and can be transmitted both vertically across generations and horizontally through parasitoid wasp parasitism, aphid feeding, or mating (Fukatsu et al. 2000). In phloem‐feeding insects such as aphids, the bacterial community within the body is dominated by obligate and facultative endosymbionts localized to insect cells or the body cavity, with very few bacteria colonizing the gut (Jing et al. 2014). Some facultative endosymbionts have been widely reported in aphids (Guo et al. 2017), such as *Serratia symbiotica* (Chen and Purcell 1997), *Hamiltonella defensa* (Darby et al. 2001), *Regiella insecticola* (Sandström et al. 2001), *Rickettsia* (Chen et al. 1996), *Rickettsiella* (Roux et al. 1997), *Fukatsuia symbiotica* (Guay et al. 2009), *Spiroplasma* (Fukatsu et al. 2001), *Wolbachia* (Werren et al. 1995), and *Arsenophonus* (Gherna et al. 1991). Although facultative symbionts are not essential for host survival and reproduction (Oliver et al. 2010), some play significant roles in regulating the ecological adaptability of aphids (Oliver et al. 2005). For example, *Serratia symbiotica* can provide aphids with a reproductive advantage following heat stress (Montllor et al. 2002); *Hamiltonella defensa* aids pea aphids in defending against parasitoid wasps (Oliver et al. 2005); and *Regiella insecticola* enhances aphid resistance to fungal pathogens (Scarborough et al. 2005).

Given the close ecological interactions between aphids and their symbiotic bacteria, this study focuses on the *Koelreuteria*‐feeding aphid species complex and employs 16S rRNA gene high‐throughput sequencing to address three key questions: (1) whether symbiont community structure significantly differs among these closely allied aphid species; (2) whether host plant species significantly influences symbiont composition in sympatric aphid populations; and (3) whether symbiont communities vary significantly among geographically distinct populations, and which climatic factors contribute most strongly to this variation. This study aims to elucidate the distribution patterns of symbiotic bacteria across host species and environmental gradients, thereby providing new evidence for understanding ecological adaptation mechanisms of close aphid species and laying a foundation for developing targeted integrated management strategies.

## 2 Methods and Materials

### 2.1 Sample Collection

**FIGURE 1:**
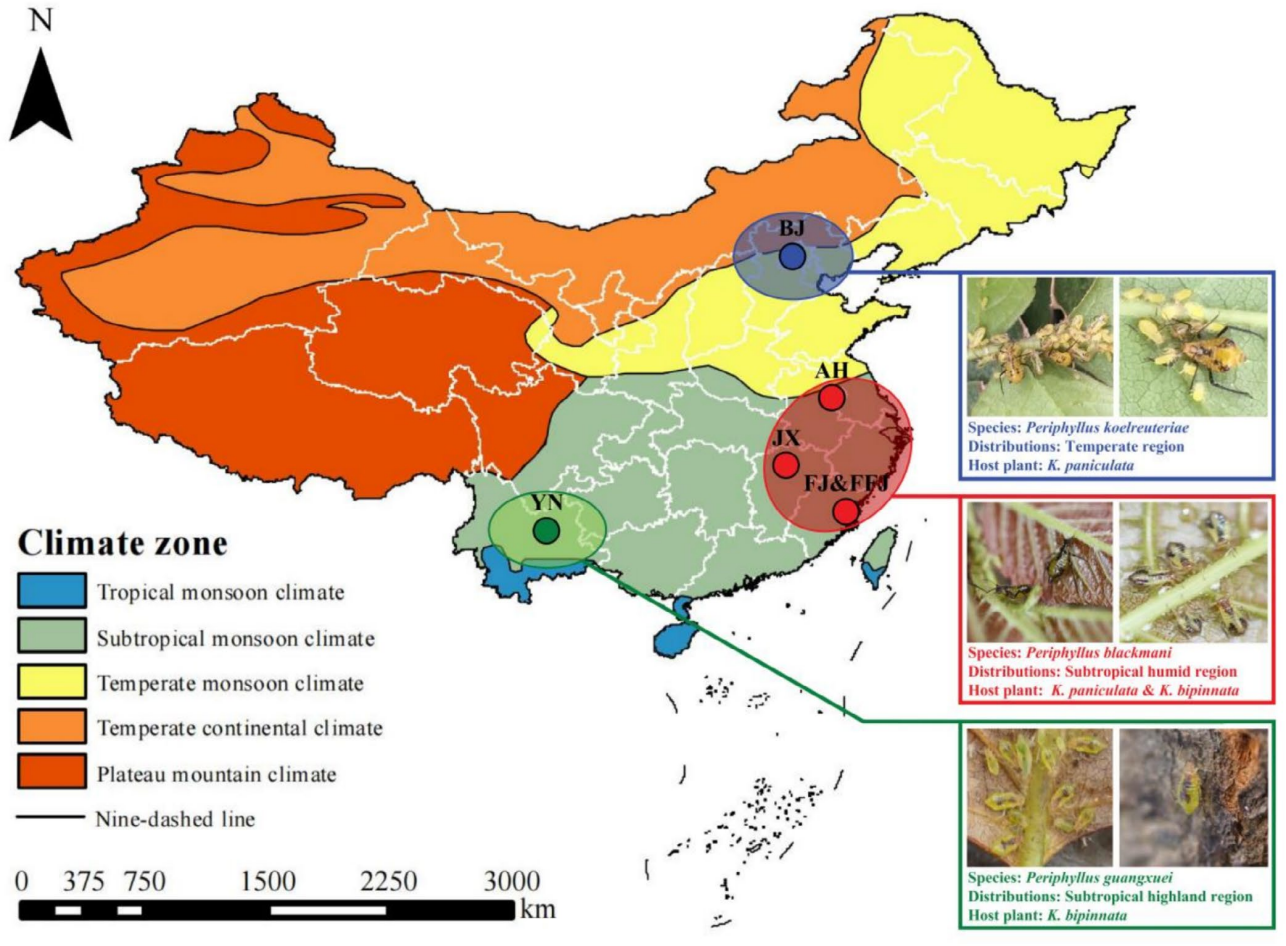
Sampling sites of *P. koelreuteriae*, *P. blackmani*, and *P. guangxuei*.

The samples of *P. koelreuteriae* were collected from Beijing (BJ), and their host plant is *Koelreuteria paniculata* . The samples of *P. blackmani* were collected from Nanchang, Jiangxi (JX); Fuzhou, Fujian (FJ & FFJ); and Chuzhou, Anhui (AH). Among these, *P. blackmani* from Fuzhou, Fujian, were sampled from two different host plants: *K. paniculata* (FJ) and *Koelreuteria bipinnata* (FFJ), whereas those from Nanchang, Jiangxi (JX), and Chuzhou, Anhui (AH), were collected exclusively from *K. paniculata* . The samples of *P. guangxuei* were collected from Kunming in Yunnan (YN), and their host plant is *K. bipinnata* (Figure 1, Table 1). All samples used in this study were wingless adult aphid individuals. Given that aphids predominantly reproduce through parthenogenesis, forming clonal populations, to ensure biological independence and avoid pseudoreplication, the three individuals collected per population were all obtained from different plants spaced more than 50 m, thereby ensuring that each individual represents a potentially distinct clone.

**TABLE 1:** *P. koelreuteriae* species complex sample collection and grouping information.

ID	Population	Species	Date	Host plants	Coordinates	Location collected
BJ1A	BJ1	*P. koelreuteriae*	2019/4/20	*K. paniculata*	40.17 N,116.288E	Beijing
BJ1B			2019/4/20	*K. paniculata*	40.17 N,116.288E	Beijing
BJ1C			2019/4/20	*K. paniculata*	40.17 N,116.288E	Beijing
BJ2A	BJ2		2019/4/20	*K. paniculata*	40.162 N,116.285E	Beijing
BJ2B			2019/4/20	*K. paniculata*	40.162 N,116.285E	Beijing
BJ2C			2019/4/20	*K. paniculata*	40.162 N,116.285E	Beijing
BJ3A	BJ3		2019/4/20	*K. paniculata*	40.16 N,116.263E	Beijing
BJ3B			2019/4/20	*K. paniculata*	40.16 N,116.263E	Beijing
BJ3C			2019/4/20	*K. paniculata*	40.16 N,116.263E	Beijing
FFJ1A	FFJ1	*P. blackmani*	2016/5/9	*K. bipinnata*	26.086 N,119.231E	Fujian, Fuzhou
FFJ1B			2016/5/9	*K. bipinnata*	26.086 N,119.231E	Fujian, Fuzhou
FFJ1C			2016/5/9	*K. bipinnata*	26.086 N,119.231E	Fujian, Fuzhou
FFJ2A	FFJ2		2017/3/28	*K. bipinnata*	26.083 N,119.234E	Fujian, Fuzhou
FFJ2B			2017/3/28	*K. bipinnata*	26.083 N,119.234E	Fujian, Fuzhou
FFJ2C			2017/3/28	*K. bipinnata*	26.083 N,119.234E	Fujian, Fuzhou
FJ1A	FJ1		2016/4/21	*K. paniculata*	26.086 N,119.23E	Fujian, Fuzhou
FJ1B			2016/4/21	*K. paniculata*	26.086 N,119.23E	Fujian, Fuzhou
FJ1C			2016/4/21	*K. paniculata*	26.086 N,119.23E	Fujian, Fuzhou
FJ2A	FJ2		2021/3/26	*K. paniculata*	26.082 N,119.235E	Fujian, Fuzhou
FJ2B			2021/3/26	*K. paniculata*	26.082 N,119.235E	Fujian, Fuzhou
FJ2C			2021/3/26	*K. paniculata*	26.082 N,119.235E	Fujian, Fuzhou
AH1A	AH1		2024/4/19	*K. paniculata*	32.337 N,118.364E	Anhui, Chuzhou
AH1B			2024/4/19	*K. paniculata*	32.337 N,118.364E	Anhui, Chuzhou
AH1C			2024/4/19	*K. paniculata*	32.337 N,118.364E	Anhui, Chuzhou
JX1A	JX1		2016/4/2	*K. paniculata*	28.666 N,115.918E	Jiangxi, Nanchang
JX1B			2016/4/2	*K. paniculata*	28.666 N,115.918E	Jiangxi, Nanchang
JX1C			2016/4/2	*K. paniculata*	28.666 N,115.918E	Jiangxi, Nanchang
JX2A	JX2		2021/5/14	*K. paniculata*	28.688 N,115.893E	Jiangxi, Nanchang
JX2B			2021/5/14	*K. paniculata*	28.688 N,115.893E	Jiangxi, Nanchang
JX2C			2021/5/14	*K. paniculata*	28.688 N,115.893E	Jiangxi, Nanchang
YN1A	YN1	*P. guangxuei*	2017/9/25	*K. bipinnata*	25.064 N,102.756E	Yunnan, Kunming
YN1B			2017/9/25	*K. bipinnata*	25.064 N,102.756E	Yunnan, Kunming
YN1C			2017/9/25	*K. bipinnata*	25.064 N,102.756E	Yunnan, Kunming
YN2A	YN2		2017/11/7	*K. bipinnata*	24.949 N,102.738E	Yunnan, Kunming
YN2B			2017/11/7	*K. bipinnata*	24.949 N,102.738E	Yunnan, Kunming
YN2C			2017/11/7	*K. bipinnata*	24.949 N,102.738E	Yunnan, Kunming

### 2.2 DNA Extraction and Cryptic Species Identification

From each geographical population, three wingless adult aphids were randomly selected. The specimens were first surface‐sterilized by immersion in 75% ethanol for 30 s, followed by three thorough rinses with ultrapure water, each lasting 5 s, to completely remove residual ethanol and surface contaminants. Then use the DNeasy Blood and Tissue DNA Extraction Kit (QIAGEN) to extract the total genomic DNA of the whole aphid samples. DNA extraction is performed in a clean bench to avoid contamination from environmental DNA. The mitochondrial COI gene sequences were amplified from all DNA samples using universal primers LCO1490 (5′‐ATTTGATCTGGAATTTTAGG‐3′) and HCO2198 (5′‐TAAACTTCAGGGTGACCAAAAA ATCA‐3′) (Folmer et al. 1994) to validate the quality of DNA extraction. Sterile deionized water was used as a negative control to ensure the reliability of the results. The PCR amplification reaction mixture of 25 μL contains 2 μL of DNA, 2.5 μL of 10 × LA PCR Buffer II (Mg^2+^ plus), 0.5 μL of dNTP mixture (2.5 mM each), 0.5 μL of each forward and reverse primer (10 μM), 0.5 μL of LA Taq enzyme (TaKaRa Bio Inc., Otsu, Japan), and 18.5 μL of ultrapure water. PCR amplification is performed using the ProFlexTM PCR system (Applied Biosystems Inc., Waltham, United States) with the following program: initial denaturation at 95°C for 2 min; 35 cycles of denaturation at 95°C for 15 s, annealing at 65°C for 15 s, and extension at 72°C for 23 s; and a final extension at 72°C for 5 min. 5 μL of PCR product was mixed with 1 μL of 6 × Loading Buffer and analyzed by 1% agarose gel electrophoresis. The remaining PCR product was sent to Biomarker Technologies Corporation for sequencing. With reference to the cryptic species identification results of *P. koelreuteriae* reported by Li et al. (2022), the cryptic species type of each sample was determined through sequence alignment. The GenBank accession numbers for the reference sequences are KX680002 for *P. koelreuteriae*, MZ396450 for *P. blackmani*, and MZ396454 for *P. guangxuei*.

### 2.3 Amplification and Sequencing of 16 s rRNA Gene

In this study, total DNA was extracted from whole aphid individuals after surface sterilization. Therefore, the extracted DNA encompassed all bacterial symbionts within the aphid body, including both intracellular endosymbiotic (e.g., *Buchnera* and *Serratia*) and potential gut bacteria. The V3 and V4 regions of the 16S ribosomal RNA gene were amplified using primers 338F (5′‐ACTCCTACGGGAGGCAGCA‐3′) and 806R (5′‐GGACTACHVGGGTWTCT AAT‐3′). Gel electrophoresis with a 1% agarose gel was performed to verify the PCR products, which were subsequently purified by gel extraction. A negative control (sterile water instead of template DNA) was included in each PCR batch and yielded no visible band on the gel, confirming the absence of reagent contamination. The 16S rRNA was then subjected to paired‐end sequencing on the Illumina NovaSeq platform at Beijing Biomarker Technologies Co. Ltd.

### 2.4 Sequencing Data Processing

In this study, the V3‐V4 hypervariable region of the bacterial 16S rRNA gene was amplified using primers 338F (5′‐ACTCCTACGGGAGGCAGCA‐3′) and 806R (5′‐GGACTACHVGGGTWTCT AAT‐3′) and sequenced on the Illumina NovaSeq platform. Raw sequencing data were initially processed with Trimmomatic (v0.33) for quality control using a sliding window approach (50 bp window size, Q20 average quality threshold). Primer sequences were then trimmed using Cutadapt (v1.9.1) with the following parameters: maximum mismatch rate of 20% and minimum primer coverage of 80%. Paired‐end reads were merged using USEARCH (v10) with the following criteria: minimum overlap length of 10 bp, 90% similarity threshold, and a maximum of 5 bp mismatches. To ensure comparability across samples, the merged high‐quality reads from all samples were rarefied (subsampled without replacement) to an even sequencing depth of 80,000 reads per sample prior to subsequent analyses. Chimeric sequences were identified and removed using UCHIME (v8.1) with default parameters, requiring query sequences to exhibit ≥ 80% similarity to both parent sequences. For microbial community analysis, we employed the DADA2 pipeline within the QIIME2 platform (v2020.6) to infer amplicon sequence variants (ASVs), using its default parameters and setting a 0.005% prevalence threshold. Taxonomic assignment was performed using a hybrid approach: sequences were first classified using classify‐consensus‐blast by selecting the most consistent taxonomy from the top three matches (minimum 90% similarity, 90% coverage, and 51% consensus threshold). Sequences without confident BLAST matches were subsequently annotated using the classify‐sklearn classifier pre‐trained on the Greengenes database (confidence threshold: 0.7). To assess sequencing depth sufficiency, alpha‐rarefaction curves were generated using the QIIME2 alpha‐rarefaction command (1000 iterations, 10% maximum depth step size). The asymptotic saturation of ASV accumulation curves confirmed that all samples reached sufficient sequencing depth (sampling saturation > 99%). The final average sequencing depth achieved was 79,296 high‐quality reads per sample.

### 2.5 Data Analysis

Alpha diversity analysis was performed on all samples using QIIME2 to calculate species richness (Chao1 index), diversity (Simpson and Shannon indices), and library coverage (Good's Coverage). The Chao1 index estimates the total number of species, with higher values indicating greater species richness. The Shannon index integrates both species richness and evenness, with higher values reflecting higher community diversity. The Simpson index focuses on the dominance of abundant species, with values approaching 1 indicating more even species distribution. Good's Coverage assesses sequencing depth adequacy, with higher values indicating more reliable data quality. Statistical differences in Shannon and Simpson indices among groups were assessed using the Kruskal–Wallis test (*p < 0.05, **p < 0.01, ***p < 0.001). Beta diversity analysis was then conducted to compare differences in symbiotic bacterial composition among different sample categories. The ASV dataset used in this study includes both presence/absence information and relative abundance information of ASVs. Community dissimilarities were quantified using Bray‐Curtis distance based on ASVs and Weighted Unifrac distance based on ASV phylogenetic information. Multivariate statistical analyses were carried out in the R v4.2.3 programming environment using the vegan v2.6.4 package. Microbial community distance matrices were constructed based on Bray‐Curtis and Weighted Unifrac distances. Non‐metric multidimensional scaling (NMDS) was applied for dimensionality reduction and visualization of samples. All NMDS analyses yielded stress values below 0.2, indicating good representation in two‐dimensional ordination. To examine significant differences in microbial communities among different groupings (species, geographic populations, and host plants), similarity analysis (ANOSIM) and permutational multivariate analysis of variance (PERMANOVA) were employed. Redundancy analysis (RDA) was used to explore the influence of environmental factors on microbial community structure. Prior to RDA, data were preprocessed using Hellinger transformation, and detrended correspondence analysis (DCA) was conducted to determine the suitability of the ordination method. During RDA, a correlation matrix among environmental factors was first calculated, and collinear variables were screened using a threshold of 0.8. The significance of each environmental factor was evaluated using 999 permutation tests. All statistical results were visualized using the ggplot2 v3.5.2 package.

## 3 Results

### 3.1 Sequencing and Classification

**FIGURE 2:**
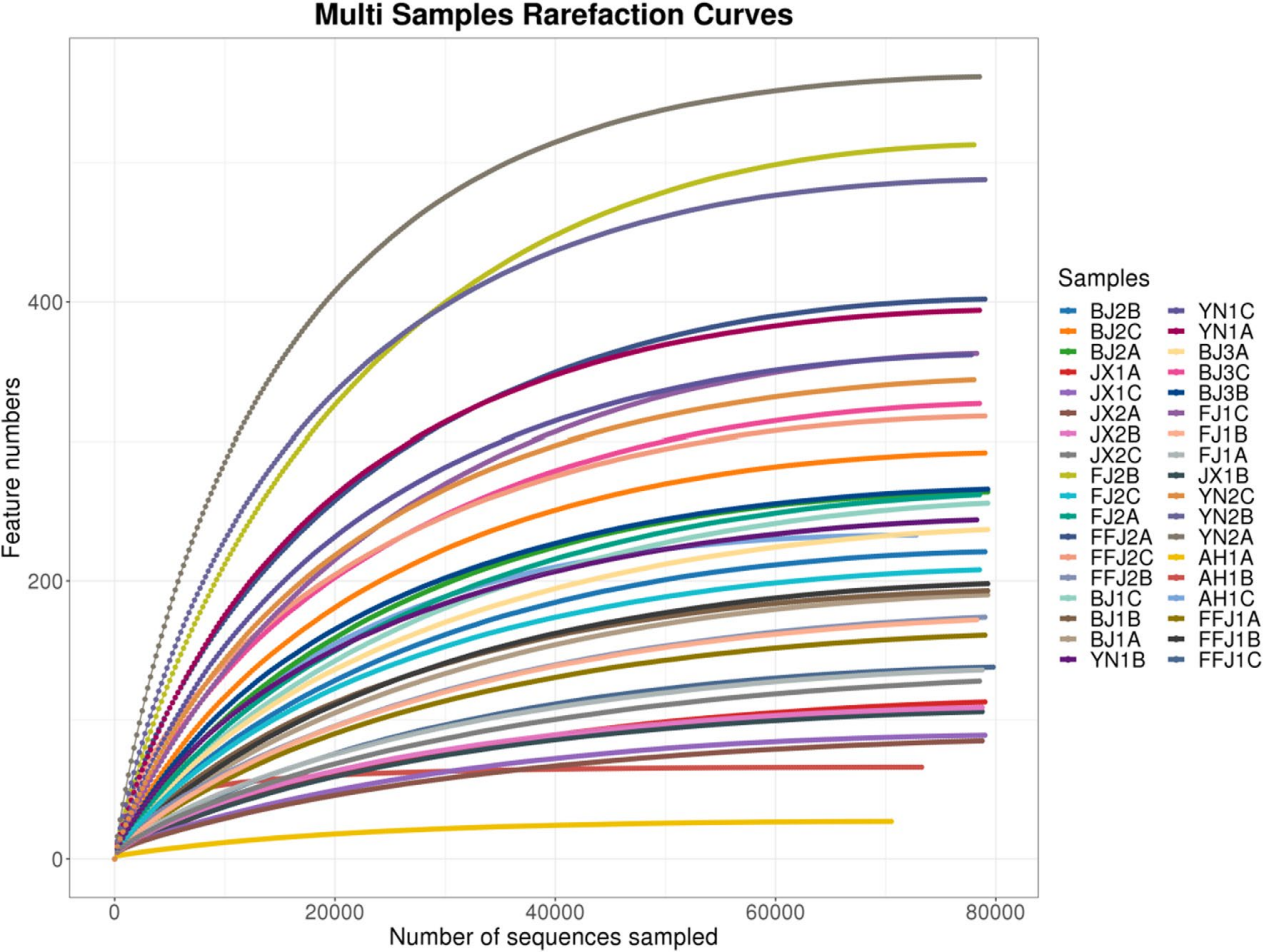
Rarefaction curves of individual samples in the *P. koelreuteriae* species complex.

Sequencing of the 16S rRNA V3–V4 region amplicons yielded a total of 2,879,833 raw reads (approximately 79,995 reads per sample on average). After quality control and chimera removal using the DADA2 algorithm, 2,827,083 valid reads were obtained (averaging approximately 78,530 valid reads per sample). Rarefaction curve analysis showed that the ASV accumulation curves for all samples reached a plateau at the current sequencing depth (Figure 2), indicating that the sequencing depth was sufficient to capture the microbial diversity present. All sequencing results were clustered into 7132 ASVs, encompassing bacteria from 37 phyla, 457 families, and 1016 genera (Table 2).

**TABLE 2:** The Illumina NovaSeq sequencing results of bacterial 16S rRNA gene.

Sample ID	Population	Species	Raw Reads	Valid Reads	ASVs	Phylum	Family	Genus
BJ1A	BJ1	*P. koelreuteriae*	80,051	79,525	190	18	90	127
BJ1B			80,260	79,669	193	16	87	125
BJ1C			80,178	79,626	256	19	126	174
BJ2A	BJ2		80,115	79,499	264	20	117	157
BJ2B			79,894	79,282	221	18	104	142
BJ2C			79,866	79,211	292	20	130	176
BJ3A	BJ3		79,986	79,486	237	18	106	154
BJ3B			80,079	79,460	266	16	113	157
BJ3C			79,543	78,880	327	21	148	207
FFJ1A	FFJ1	*P. blackmani*	79,975	79,178	161	15	82	110
FFJ1B			79,941	79,423	198	16	104	140
FFJ1C			80,510	80,021	138	14	76	105
FFJ2A	FFJ2		80,148	79,251	402	25	169	235
FFJ2B			80,066	79,399	174	18	83	112
FFJ2C			80,149	79,397	318	18	121	174
FJ1A	FJ1		79,992	79,040	136	14	69	102
FJ1B			79,879	78,449	172	13	88	117
FJ1C			79,691	78,709	363	21	150	223
FJ2A	FJ2		80,384	78,719	262	20	115	167
FJ2B			79,895	78,354	513	23	195	299
FJ2C			79,985	78,712	208	16	93	142
AH1A	AH1		79,848	70,770	27	8	19	20
AH1B			79,745	73,359	66	9	38	47
AH1C			79,998	73,057	233	22	111	156
JX1A	JX1		80,086	79,364	113	15	67	88
JX1B			80,077	79,085	106	13	56	76
JX1C			80,072	79,251	89	11	46	60
JX2A	JX2		80,161	79,088	85	11	50	63
JX2B			79,808	79,135	109	13	57	79
JX2C			79,740	78,810	128	15	73	89
YN1A	YN1	*P. guangxuei*	80,031	78,762	394	22	143	209
YN1B			79,751	78,612	244	15	104	147
YN1C			80,033	78,076	362	20	142	217
YN2A	YN2		79,742	78,803	562	24	175	270
YN2B			80,287	79,391	488	20	161	248
YN2C			79,867	78,230	344	18	137	200
Total			2,879,833	2,827,083	7132	37	457	1016

### 3.2 Analysis of Bacterial Community Composition and Abundance

**FIGURE 3:**
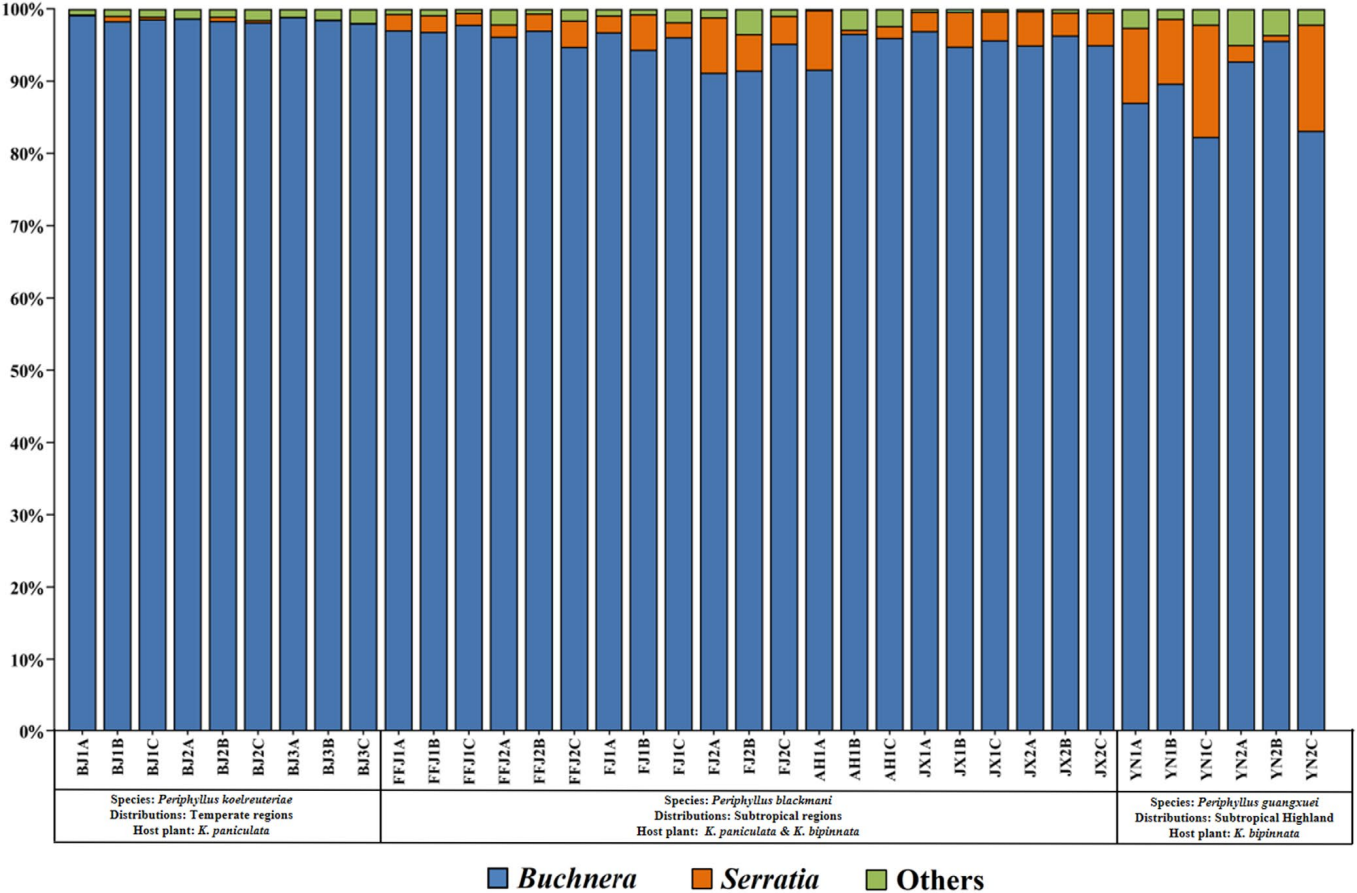
Taxonomic composition and relative abundance of symbiotic bacteria at genus level for all *P. koelreuteriae*, *P. blackmani*, and *P. guangxuei* samples.

At the bacterial genus level, the primary symbiont *Buchnera* was overwhelmingly dominant in almost all samples, with an average relative abundance of 94.99%. The only other genus with an average relative abundance exceeding 1% was *Serratia* (3.60%). The mean relative abundance of *Buchnera* exhibited the following trend: *P. guangxuei* (88.41%) < *P. blackmani* (95.36%) < *P. koelreuteriae* (98.51%). In contrast, the mean relative abundance of *Serratia* showed the opposite pattern: *P. guangxuei* (8.79%) > *P. blackmani* (3.55%) > *P. koelreuteriae* (0.27%) (Figure 3, Table 3).

**TABLE 3:** Symbiotic bacterial composition of individual samples in the *P. koelreuteriae* species complex.

Samples ID	Population	Species	*Buchnera*	*Serratia*	Others
BJ1A	BJ1	*P. koelreuteriae*	78,650 (99.14%)	95 (0.12%)	587 (0.74%)
BJ1B			78,137 (98.30%)	612 (0.77%)	736 (0.93%)
BJ1C			78,280 (98.59%)	270 (0.34%)	846 (1.07%)
BJ2A	BJ2		78,232 (98.67%)	32 (0.04%)	1023 (1.29%)
BJ2B			77,790 (98.36%)	473 (0.60%)	825 (1.04%)
BJ2C			77,562 (98.16%)	275 (0.35%)	1182 (1.50%)
BJ3A	BJ3		78,432 (98.88%)	43 (0.05%)	846 (1.07%)
BJ3B			78,065 (98.49%)	53 (0.07%)	1147 (1.45%)
BJ3C			77,101 (98.02%)	52 (0.07%)	1503 (1.91%)
FFJ1A	FFJ1	*P. blackmani*	76,672 (97.05%)	1798 (2.28%)	530 (0.67%)
FFJ1B			76,768 (96.84%)	1846 (2.33%)	655 (0.83%)
FFJ1C			78,105 (97.80%)	1343 (1.68%)	418 (0.52%)
FFJ2A	FFJ2		75,999 (96.15%)	1372 (1.74%)	1669 (2.11%)
FFJ2B			76,873 (97.01%)	1898 (2.40%)	473 (0.60%)
FFJ2C			75,066 (94.78%)	2886 (3.64%)	1251 (1.58%)
FJ1A	FJ1		76,308 (96.76%)	1849 (2.34%)	706 (0.90%)
FJ1B			73,834 (94.36%)	3871 (4.95%)	545 (0.70%)
FJ1C			75,314 (96.09%)	1642 (2.09%)	1423 (1.82%)
FJ2A	FJ2		71,583 (91.17%)	6032 (7.68%)	901 (1.15%)
FJ2B			71,414 (91.49%)	3937 (5.04%)	2706 (3.47%)
FJ2C			74,729 (95.18%)	3024 (3.85%)	758 (0.97%)
AH1A	AH1		64,816 (91.63%)	5834 (8.25%)	89 (0.13%)
AH1B			70,825 (96.57%)	426 (0.58%)	2091 (2.85%)
AH1C			69,990 (96.01%)	1183 (1.62%)	1722 (2.36%)
JX1A	JX1		76,789 (96.94%)	2108 (2.66%)	314 (0.40%)
JX1B			74,847 (94.80%)	3805 (4.82%)	301 (0.38%)
JX1C			75,702 (95.65%)	3202 (4.05%)	238 (0.30%)
JX2A	JX2		74,991 (94.95%)	3777 (4.78%)	212 (0.27%)
JX2B			76,091 (96.34%)	2528 (3.20%)	363 (0.46%)
JX2C			74,724 (95.01%)	3553 (4.52%)	374 (0.48%)
YN1A	YN1	*P. guangxuei*	68,383 (87.02%)	8151 (10.37%)	2047 (2.60%)
YN1B			70,313 (89.67%)	7050 (8.99%)	1051 (1.34%)
YN1C			64,076 (82.29%)	12,110 (15.55%)	1683 (2.16%)
YN2A	YN2		72,948 (92.76%)	1805 (2.30%)	3891 (4.95%)
YN2B			75,727 (95.59%)	638 (0.81%)	2854 (3.60%)
YN2C			64,882 (83.14%)	11,495 (14.73%)	1667 (2.14%)

### 3.3 Comparison of Symbiotic Bacterial Communities Among Different Geographic Populations, Aphid Species, and Host Plants

**FIGURE 4:**
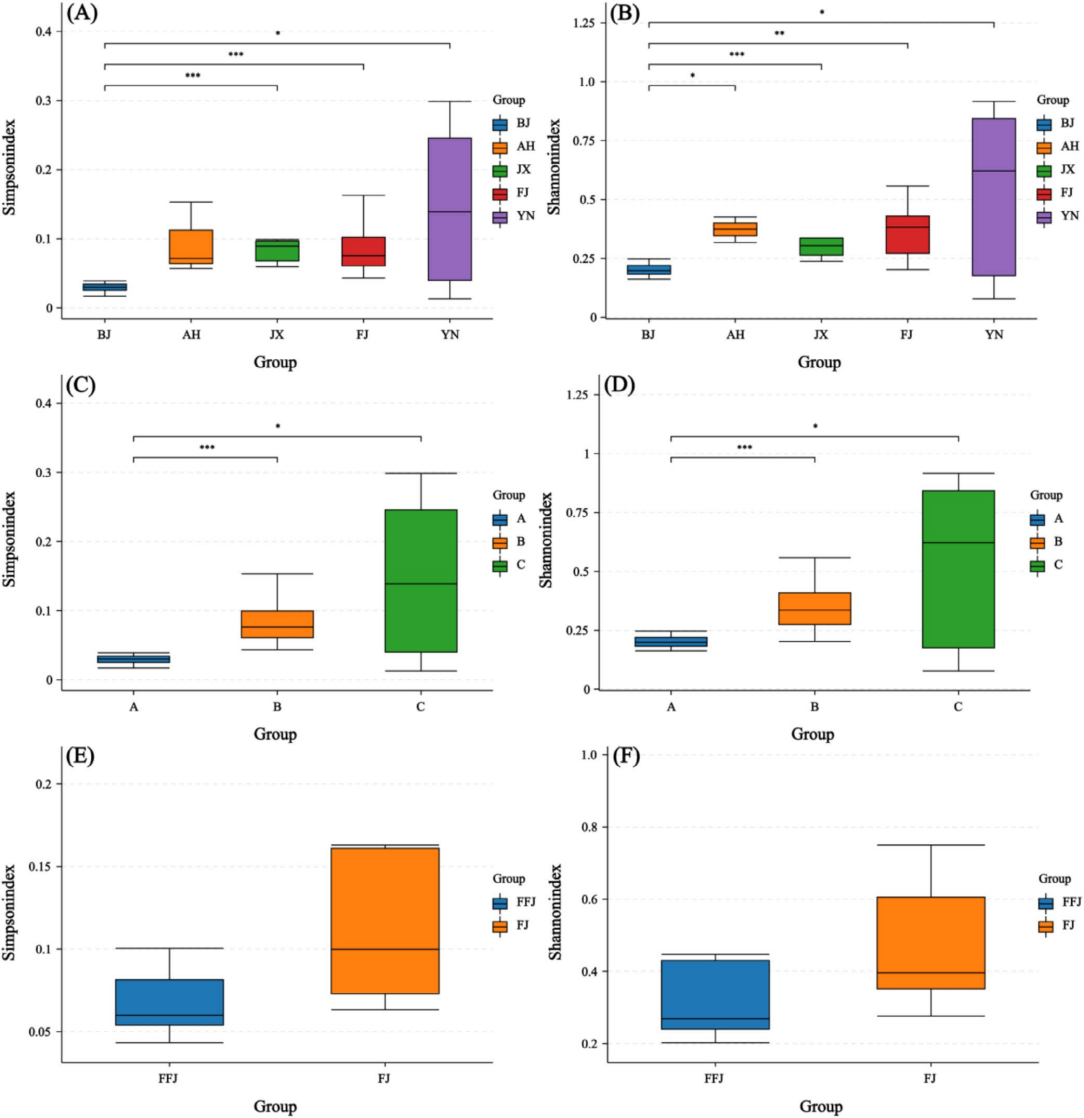
Boxplots of symbiont alpha diversity (Shannon index (A) & Simpson (B)) for three aphid species (*P. koelreuteriae*, *P. blackmani*, and *P. guangxuei*); Boxplots of symbiont alpha diversity (Shannon index (C) & Simpson (D)) for five groups of geographic regions (BJ, FFJ, FJ, JX, YN); Boxplots of symbiont alpha diversity (Shannon index (E) & Simpson (F)) for *P. blackmani* host plant groups feeding on *K. bipinnata* (FFJ) and *K. paniculata* (FJ).

The alpha diversity of bacterial communities within each aphid sample was assessed using four metrics: Chao1, Shannon, Simpson, and Coverage. Across all samples, the coverage values for the sampling completeness indicator were all > 99%, indicating that the sampling method used was capable of detecting sufficient bacterial diversity (Table 4). The Kruskal‐Wallis test at the species level revealed significant differences in the alpha diversity of symbiotic bacterial communities among aphid species (Figure 4). Specifically, *P. koelreuteriae* exhibited significantly lower alpha diversity (Shannon: mean = 0.52, SD = 0.05; Simpson: mean = 0.12, SD = 0.01) compared to both *P. blackmani* (Shannon: mean = 0.66, SD = 0.17; Simpson: mean = 0.17, SD = 0.04) and *P. guangxuei* (Shannon: mean = 1.08, SD = 0.14; Simpson: mean = 0.28, SD = 0.07), indicating that the symbiotic bacterial community of *P. koelreuteriae* is structurally simpler with more dominant species. In contrast, no significant difference was observed between *P. blackmani* and *P. guangxuei*, suggesting that their symbiotic bacterial communities have similar levels of complexity. At the population level, Kruskal‐Wallis tests revealed significant differences in the alpha diversity of symbiotic bacterial communities among geographical populations. Both Shannon and Simpson indices indicated that the BJ population exhibited the lowest bacterial community diversity and the dominance of dominant species is most prominent. Specifically, the BJ population exhibited significantly lower alpha diversity (Shannon: mean = 0.52, SD = 0.05; Simpson: mean = 0.12, SD = 0.01) compared to the FJ (Shannon: mean = 0.73, SD = 0.17; Simpson: mean = 0.18, SD = 0.03), JX (Shannon: mean = 0.64, SD = 0.04; Simpson: mean = 0.19, SD = 0.02), and YN (Shannon: mean = 1.08, SD = 0.14; Simpson: mean = 0.28, SD = 0.07) populations. Notably, the Shannon index, being more sensitive to rare species, detected subtle differences in community evenness between the BJ and AH populations, whereas this difference was not reflected in the Simpson index, which focuses more on dominant species. At the host plant level, to exclude the effects of aphid species and geographical populations, we only compared the symbiotic bacterial community composition and alpha diversity of sympatrically distributed *P. blackmani* (populations FJ and FFJ). Both the Shannon index and Simpson index showed no significant differences in the community composition or alpha diversity of symbiotic bacteria in *P. blackmani* feeding on *K. paniculata* (Shannon: mean = 0.76, SD = 0.16; Simpson: mean = 0.19, SD = 0.03) and *K. bipinnata* (Shannon: mean = 0.69, SD = 0.19; Simpson: mean = 0.17, SD = 0.03).

**TABLE 4:** The bacteria Alpha diversity index for aphid samples.

Sample ID	Type	Chao1	Simpson	Shannon	Coverage
BJ1A	BJ1	192.59	0.11	0.46	1.00
BJ1B		194.84	0.12	0.5	1.00
BJ1C		259.95	0.12	0.51	1.00
BJ2A	BJ2	264.63	0.11	0.5	1.00
BJ2B		222.14	0.13	0.52	1.00
BJ2C		293.05	0.12	0.57	1.00
BJ3A	BJ3	241.15	0.12	0.49	1.00
BJ3B		267.63	0.12	0.53	1.00
BJ3C		328.86	0.14	0.63	1.00
FFJ1A	FFJ1	164.79	0.15	0.56	1.00
FFJ1B		200.44	0.15	0.59	1.00
FFJ1C		139.52	0.14	0.52	1.00
FFJ2A	FFJ2	402.57	0.18	0.89	1.00
FFJ2B		178.78	0.15	0.61	1.00
FFJ2C		318.58	0.22	0.98	1.00
FJ1A	FJ1	139.68	0.16	0.6	1.00
FJ1B		176.05	0.19	0.69	1.00
FJ1C		364.01	0.16	0.7	1.00
FJ2A	FJ2	265.6	0.24	0.84	1.00
FJ2B		513.73	0.23	1.05	1.00
FJ2C		210.47	0.18	0.68	1.00
AH1A	AH	27.00	0.15	0.43	1.00
AH1B		66.00	0.07	0.39	1.00
AH1C		233.00	0.08	0.43	1.00
JX1A	JX1	117.13	0.17	0.6	1.00
JX1B		109.58	0.2	0.69	1.00
JX1C		89.90	0.19	0.66	1.00
JX2A	JX2	89.39	0.2	0.66	1.00
JX2B		110.77	0.16	0.59	1.00
JX2C		135.47	0.19	0.66	1.00
YN1A	YN1	395.47	0.31	1.16	1.00
YN1B		246.85	0.26	0.93	1.00
YN1C		362.98	0.36	1.21	1.00
YN2A	YN2	562.65	0.22	1.15	1.00
YN2B		488.67	0.17	0.89	1.00
YN2C		346.28	0.34	1.15	1.00
Average		242.23	0.18	0.70	1.00

**FIGURE 5:**
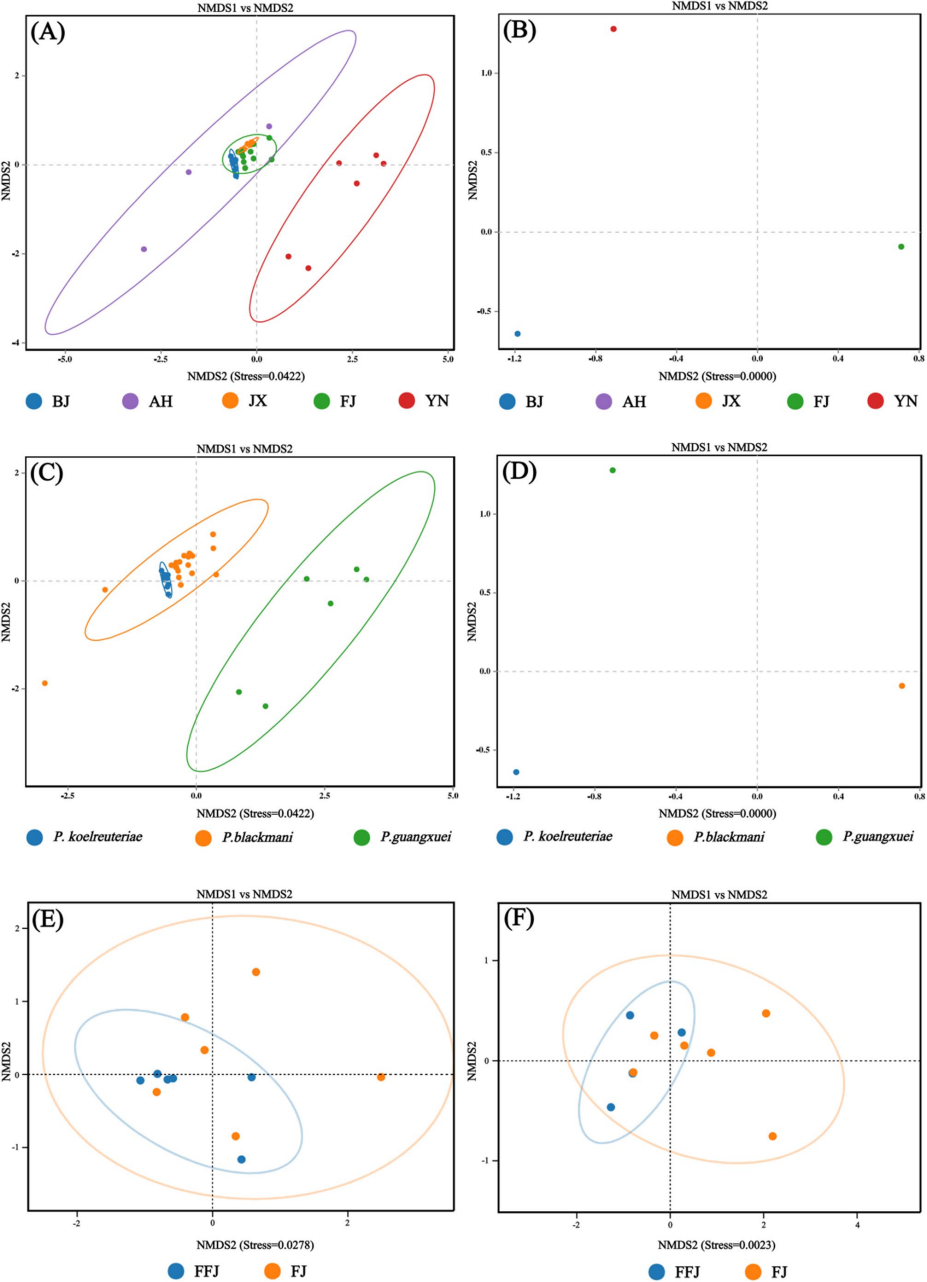
NMDS plots of symbiotic bacterial communities based on weighted UniFrac (A) and Bray‐Curtis (B) for three aphid species (*P. koelreuteriae*, *P. blackmani* and *P. guangxuei*); NMDS plots based on weighted UniFrac (C) and Bray‐Curtis (D) for five groups of geographic regions (BJ, FFJ, FJ, JX, YN); NMDS plots based on weighted UniFrac (E) and Bray‐Curtis (F) for *P. blackmani* host plant groups feeding on *K. bipinnata* (FFJ) and *K. paniculata* (FJ).

NMDS plots revealed separation trends of symbiotic bacterial communities among different geographical populations, species, and host plants of aphids (Figure 5). Specifically, NMDS plots for different geographical populations and species showed three distinct clusters, while no clear separation trend was observed for different host plants. ANOSIM and PERMANOVA results based on Weighted Unifrac and Bray‐Curtis distances indicated significant differences in symbiotic bacterial communities among different aphid species (ANOSIM: *R* = 0.628 [W] & 0.831 [B], *p* < 0.001; PERMANOVA: *R* ^2^ = 0.677 [W] & 0.995 [B], *p* < 0.001) and among different geographical populations (ANOSIM: *R* = 0.569 [W] & 1.000 [B], *p* < 0.001; PERMANOVA: *R* ^2^ = 0.592 [W] & 0.994 [B], *p* < 0.01). In contrast, no significant differences were found in the symbiotic bacterial communities of *P. blackmani* from different host plants (ANOSIM: *R* = 0.024 [W] & 0.174 [B], *p* > 0.05; PERMANOVA: *R* ^2^ = 0.141 [W] & 0.243 [B], *p* > 0.05) (Table 5).

**TABLE 5:** Results of ANOSIM and PERMANOVA based on Bray–Curtis and Weighted Unifrac distances.

Groups	Weighted Unifrac		Bray‐Curtis	
	ANOSIM (*R*, *p*)	PERMANOVA (*R* ^2^, *p*)	ANOSIM (*R*, *p*)	PERMANOVA (*R* ^2^, *p*)
Aphid species	0.628, 0.001	0.677, 0.001	0.831, 0.001	0.995, 0.001
Geography	0.569, 0.001	0.592, 0.001	1.000, 0.001	0.994, 0.001
Host	0.024, 0.318	0.141, 0.182	0.174, 0.079	0.243, 0.051

### 3.4 Influence of Environmental Conditions From Different Geographical Regions on the Structure of Symbiotic Bacterial Communities

**FIGURE 6:**
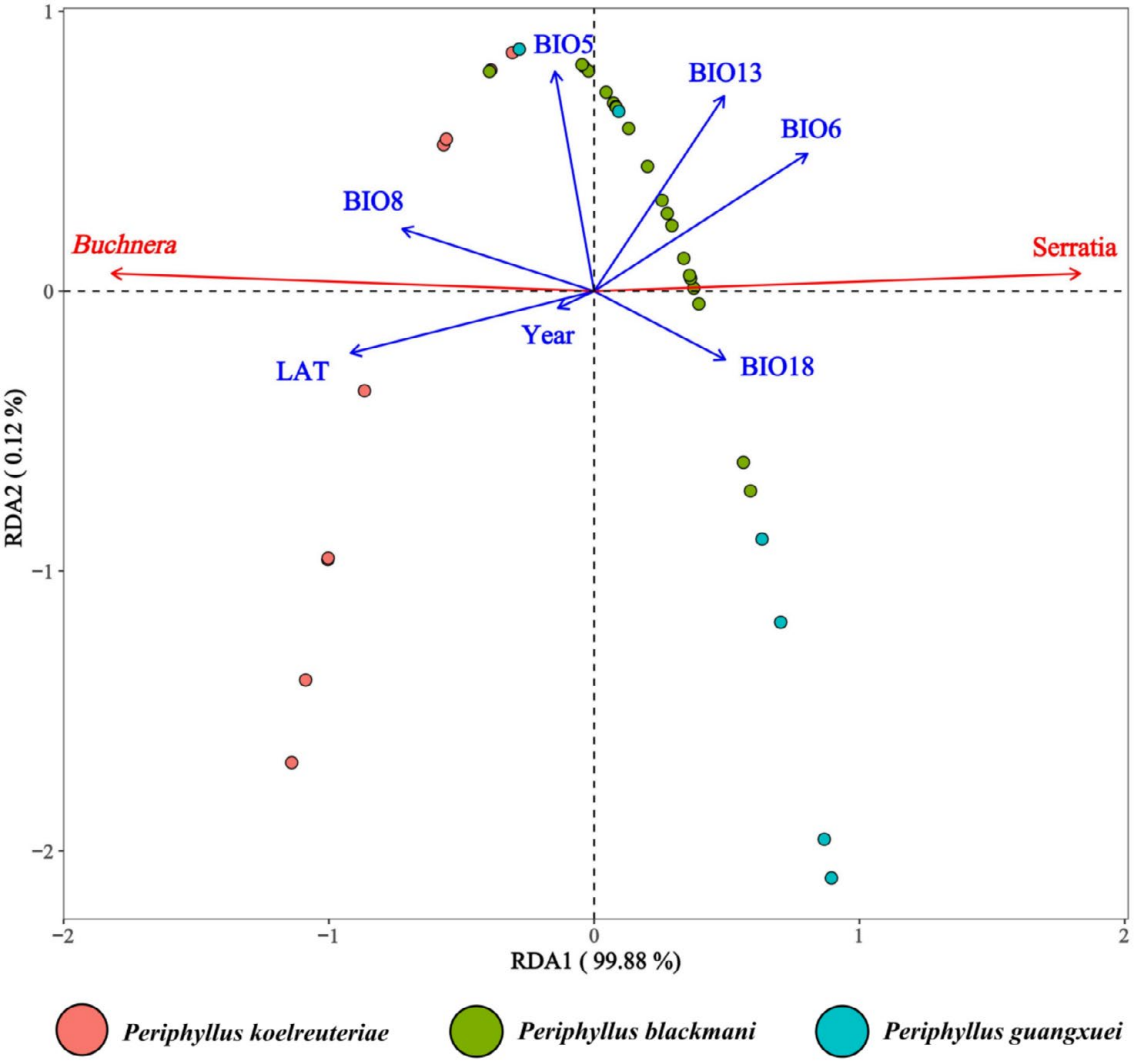
Redundancy analysis (RDA) for the relationships of environmental factors and symbiont abundance in the *P. koelreuteriae* species complex.

Results from redundancy analysis (RDA) showed that the first constrained axis (RDA1) explained 99.88% of the community variation (Figure 6). The relative abundance of *Buchnera* was positively correlated with latitude (LAT), sampling year (Year), mean temperature of the wettest quarter (BIO8), and maximum temperature of the warmest month (BIO5), while negatively correlated with minimum temperature of the coldest month (BIO6), precipitation of the warmest quarter (BIO18), and precipitation of the wettest month (BIO13). In contrast, the relative abundance of *Serratia* exhibited an opposite correlation pattern. Permutation tests indicated that all examined factors, except for the sampling year (Year, *R* ^2^ = 0.017, *p* = 0.754), significantly influenced the microbial community structure. Among these factors, latitude (LAT, *R* ^2^ = 0.706, *p* = 0.001) and minimum temperature of the coldest month (BIO6, *R* ^2^ = 0.622, *p* = 0.001) exhibited the highest explanatory power, suggesting that latitudinal gradient and low‐temperature stress are key drivers of variation in the symbiotic bacterial community. In addition, mean temperature of the wettest quarter (BIO8, *R* ^2^ = 0.424, *p* = 0.002) and precipitation of the wettest month (BIO13, *R* ^2^ = 0.368, *p* = 0.001) also showed significant effects, while precipitation of the warmest quarter (BIO18, *R* ^2^ = 0.207, *p* = 0.023) and maximum temperature of the warmest month (BIO5, *R* ^2^ = 0.204, *p* = 0.026) had a relatively weaker yet statistically significant influence. These results confirm that the composition of the symbiotic bacterial community in this species complex is primarily shaped by latitudinal gradients, temperature extremes (particularly the winter low temperature), and seasonal precipitation patterns, with latitude and the minimum temperature of the coldest month having the most prominent effects.

## 4 Discussion

This study analyzed the bacterial communities of *P. koelreuteriae*, *P. blackmani*, and *P. guangxuei*, and found that the bacterial community composition of these three species is relatively simple, mainly consisting of the dominant symbiont *Buchnera* (mean abundance: 94.99%) and the facultative symbiont *Serratia* (mean abundance: 3.60%). This simplified community structure is similar to the characteristic *Buchnera*‐*Serratia* dual symbiotic system reported by Monnin et al. (2020) in *Periphyllus* aphids. During genomic reduction, *Buchnera* first lost genes for vitamin B_2_ synthesis, followed by those for histidine and tryptophan. Meanwhile, *Serratia* gradually took over the lost riboflavin (vitamin B_2_) synthesis function, consequently forming a metabolic complementation mechanism. Moreover, the host's dependence on *Serratia* increases with the duration of symbiotic history. In this study, the relative abundance of *Serratia* varied among aphid species: *P. guangxuei* (8.79%) > *P. blackmani* (3.55%) > *P. koelreuteriae* (0.27%). This variation may be associated with the evolutionary transfer of metabolic functions within the *Buchnera*‐*Serratia* dual symbiotic system. The higher abundance in *P. guangxuei* could suggest a more advanced functional integration of *Serratia* in its symbiotic system, whereas the lower abundance in other species may reflect an earlier developmental stage of the symbiotic relationship. The compositional features of the *Buchnera*‐*Serratia* system observed in this species complex provide a potential research model for further investigation into the dynamic process of aphid‐symbiont coevolution.

Alpha diversity analysis revealed that *P. koelreuteriae* exhibited significantly lower Shannon and Simpson indices than the other two aphid species, reflecting a simpler community structure with more dominant species, likely due to its lower *Serratia* abundance and greater dependence on *Buchnera*. At the population level, the Beijing population showed the lowest diversity; the Shannon index detected significant differences between the Beijing and Anhui populations, whereas the Simpson index, which primarily reflects the contribution of dominant species, did not. This suggests that the differences between the two populations may mainly lie in rare species or community evenness. This finding highlights the complementarity of the two indices in assessing different dimensions of community structure. No significant alpha diversity differences were found in *P. blackmani* feeding on different host plants, indicating that its symbiotic bacterial community may be insensitive to host switching. Beta diversity analysis further confirmed significant separation in community composition among aphid species and geographical populations, but not among host plants. These results indicate that host species identity and geographical isolation are the primary drivers of symbiotic bacterial community structure, with host plants playing a relatively limited role. This may be related to the fact that the *Koelreuteria* plants fed upon by these aphid species are relatively closely related, with relatively small differences in secondary metabolites.

Symbiotic bacteria play a crucial role in the survival, development, and adaptation of aphids. Among them, the obligate endosymbiont *Buchnera* constitutes the physiological foundation of aphid adaptation (Baumann 2005), whereas facultative symbionts, though not essential for survival and reproduction, provide various important ecological functions. For instance, they can enhance resistance against pathogenic fungi and parasitoid wasps, improve heat tolerance, and assist in regulating adaptation to different host plants (Csorba et al. 2024; Gimmi and Vorburger 2024; Guo et al. 2017). Recent studies on the black cowpea aphid further indicate that climatic conditions significantly shape its symbiotic bacterial community structure. In humid subtropical climates, facultative symbionts (especially *Serratia symbiotica* ) are most abundant and can partially compensate for functional deficiencies of *Buchnera*. In contrast, in cold semi‐arid or tropical desert climates, facultative symbionts are scarce or even absent, leading to a significant decline in aphid adaptability when *Buchnera* is eliminated (Heidari Latibari et al. 2025). Given the extremely low abundance and limited functional contribution of gut bacteria in phloem‐feeding aphids, this study focuses its analysis on the major intracellular endosymbiotic, *Buchnera* and *Serratia*. A similar pattern was observed in this study on the *Koelreuteria*‐feeding aphid species complex. Populations distributed in subtropical humid regions and subtropical plateau (e.g., Kunming) exhibited significantly higher relative abundances of facultative symbionts. In contrast, populations in temperate regions (e.g., Beijing) showed notably lower abundances. These findings support the hypothesis that climatic conditions indirectly regulate aphid ecological adaptation strategies to different habitat challenges by shaping symbiotic bacterial community structures.

However, it is important to note that the sampling in this study has limitation—each climatic type corresponds to only one aphid species: *P. koelreuteriae* is exclusively distributed in temperate regions (Beijing), *P. blackmani* is confined to subtropical humid regions, and *P. guangxuei* is only found in subtropical highlands (Kunming). This complete spatial overlap makes it statistically challenging to separate the variables “climatic type” and “species identity.” Therefore, the observed variations in symbiotic bacterial communities may result from the dual drivers of species divergence and geographic‐climatic conditions, which collectively shape the symbiotic bacterial community structure of this complex. To disentangle the independent and interactive effects of host and climatic factors, future studies should integrate approaches such as transplanting the same aphid species across different climatic regions and common garden experiments to systematically elucidate their respective contributions and synergistic influences on symbiotic bacterial community composition.

Although host plants are widely recognized as important factors shaping the symbiotic bacterial communities of aphids (Ferrari et al. 2012; Gauthier et al. 2015; Xu et al. 2020), this study did not detect a significant effect of host plants on the structure of symbiotic bacterial communities. This result may also be related to the limitation of the experimental design: the assessment of host plant effects was based solely on a comparison of sympatrically distributed *P. blackmani* on two host plant species. While this design helps control for confounding geographic and species‐related factors, the limited sample size and host plant types may not have fully captured the ecological role of host plants. Future studies should conduct systematic comparisons across a broader range of host plant types and geographical distributions to further clarify the role of host plants in shaping aphid‐symbiont interactions.

RDA analysis indicated that latitude and the minimum temperature of the coldest month are key environmental factors influencing the symbiotic bacterial composition of this species complex. The relative abundance of *Serratia* was significantly higher in regions with lower latitudes and higher minimum temperatures of the coldest month, suggesting that the alleviation of low‐temperature stress during winter may serve as an important selective pressure driving the adaptive differentiation of symbiotic bacteria. As the primary symbiont responsible for nutrient provision, *Buchnera* exhibits a limited direct response to temperature changes. We speculate that the increase in *Serratia* abundance may represent an adaptive strategy of the host to cope with changes in winter low‐temperature environments. Future research could use this aphid‐symbiont system as a model to further elucidate the specific regulatory mechanisms of *Serratia* in the temperature adaptation process.

## 5 Conclusion

In summary, this study, based on 16S rRNA gene sequencing, elucidated the composition and relative abundance of symbiotic bacteria in three closely allied aphid species (*P. koelreuteriae*, *P. blackmani*, and *P. guangxuei*) that feed on *Koelreuteria* plants. The results showed that all three aphid species exhibit a typical dual symbiotic system dominated by *Buchnera* as the obligate symbiont, with *Serratia* as the facultative symbiont, and their relative abundance varied significantly among species and across climatic zones. This indicates that the symbiotic bacterial community structure is shaped jointly by species differentiation and geographic‐climatic conditions. Among the environmental factors, latitude (LAT) and the minimum temperature of the coldest month (BIO6) were identified as the most significant drivers influencing the composition of symbiotic bacteria. This study reveals the geographic distribution patterns of the symbiotic bacterial community in a species complex and its environmental driving factors, providing a foundation for further elucidating its ecological adaptation mechanisms and formulating targeted pest control strategies.

## Author Contributions

**Xin Peng:** conceptualization (equal), data curation (equal), methodology (equal), writing – original draft (equal), writing – review and editing (equal). **Qian Liu:** formal analysis (equal), investigation (equal), methodology (equal), software (equal), supervision (equal). **Qiang Li:** data curation (equal), methodology (equal), software (equal). **Xiaolei Huang:** formal analysis (equal), funding acquisition (equal), writing – review and editing (equal).

## Funding

This work was supported by the National Natural Science Foundation of China (32270499). National Science and Technology Fundamental Resources Investigation Program of China (2022FY100500).

## Conflicts of Interest

The authors declare no conflicts of interest.

## Data Availability

The data supporting the findings of this study are openly available in the NCBI database. All 16S rRNA gene amplicon sequencing raw data are available under the BioProject accession number PRJNA1025156.
